# Users' passivity in accessing digested scientific evidence through social media: cross-sectional insights

**DOI:** 10.1186/s13104-022-06089-x

**Published:** 2022-06-23

**Authors:** Gabriela M. Machado, Mariana M. Braga

**Affiliations:** Orthodontics and Pediatric Dentistry Department, Faculty of Dentistry, University of São Paulo, Av. Prof. Lineu Prestes, 2227 - Butantã, São Paulo, SP 05508-000 Brazil

**Keywords:** Evidence-based dentistry, Social media*, Mobile applications, Translational research

## Abstract

**Objectives:**

This manuscript provides novel insights about the potential use of social media (a Facebook page, the first strategic attempt by EviDent initiative) to share evidence-based dentistry content and empowerment strategies for professionals, using quantifiable usage metrics, besides exposing the strengths and weaknesses of this knowledge translation strategy. One year-long gathered metrics were analyzed to understand information about usage patterns.

**Results:**

Publications were potentially exposed to 4784 users, and subsequent interaction with the page occurred in 18% of cases. Users' involvement with page content was associated with the number of page visitors (P = .005). However, users' interaction with the page was not associated with the potential number of users that could have seen the page (P = .25). Even considering the users that approved the posts, only 7%, on average, interacted with the post's links. Although social media has effectively disseminated scientific content, our experience revealed the user's passivity in interacting with the content. We expect to overcome these barriers by developing a mobile app to offer a more interactive and dynamic interface associated with a more attractive format for posting, including images and infographics.

**Supplementary Information:**

The online version contains supplementary material available at 10.1186/s13104-022-06089-x.

## Introduction

Social media has been shown as a feasible strategy to disseminate scientific evidence to professionals [1–3]. Nevertheless, its use for this purpose is still limited. Credibility seems to be the critical factor when obtaining any information on the internet, including scientific evidence [4–6]. High-quality information has been sought in social media through professional communities [3, 7] or experts or renowned organizations [8].

The EviDent initiative (https://evident.fo.usp.br) to offer digested scientific content based on reliable evidence in Pediatric Dentistry has been proposed in the end of 2013 by researchers from the Dental School, University of São Paulo [9]. This initiative's primary purpose was to spread reliable and "digested" scientific content, usually found in scientific publications, to dentists and dental professionals [10].

Besides, the page pioneered in its field due to innovations in the translational process. A systematic process of scientifically digesting the evidence was created to avoid the potential inclusion of bias [11]. Since autonomy is also of utmost importance to evidence-based practice, an additional long-term purpose is to empower users to deeply understand the scientific methodology and analyze future evidence autonomously [10].

Health professionals have been already using social media to be updated and interact with scientific evidence [2, 3, 12]. Although some studies have suggested some users' passive interaction with social media [13–16], the health professionals' patterns of interaction with scientific digested contents are still underexplored.

This manuscript provides novel insights about the potential use of social media to share evidence-based dentistry content and empowerment strategies for professionals, using quantifiable usage metrics, besides exposing the strengths and weaknesses of this knowledge translation strategy.

## Main text

### Methods

This is a cross-sectional analytical study investigating 1-year data of users' interaction with digested scientific content posted on a Facebook page. The topics and dissemination of the page focused on Pediatric Dentistry; however, the page was open to any user.

#### Digested content and users' empowerment—study setting

The EviDent initiative is affiliated with the Pediatric Dentistry Department, Dental School, University of São Paulo and is led by Pediatric Dentistry researchers willing to disseminate evidence-based practice. These researchers manage a group of graduate and undergraduate students responsible for scientific and technical support for the products derived from this initiative. The Facebook Page named: "Odontopediatria: Evidências para você!" (Pediatric Dentistry: evidence for you!) was created in 2013 [10] as the first milestone in the history of EviDent. The page was initially available in Brazilian Portuguese and Facebook was chosen as the social media to disseminate the idea since, on that occasion, it was the most used one among Brazilians.

The EviDent staff met periodically to choose relevant topics for dentists' clinical practice (Additional file 1). Afterwards, the staff prepared a brief digested content related to a piece of evidence and additional content to stimulate professionals' awareness [17] and empowerment for digesting evidence by themselves. For that, researchers created a systematic process to avoid a biased report and permit reproducibility of the strategy [10].

A standard structure for the posts was also defined for the page (Additional file 2), including symbols to help clinicians understand digested evidence's relevance (Additional file 3, Fig. 1).

**Fig. 1:**
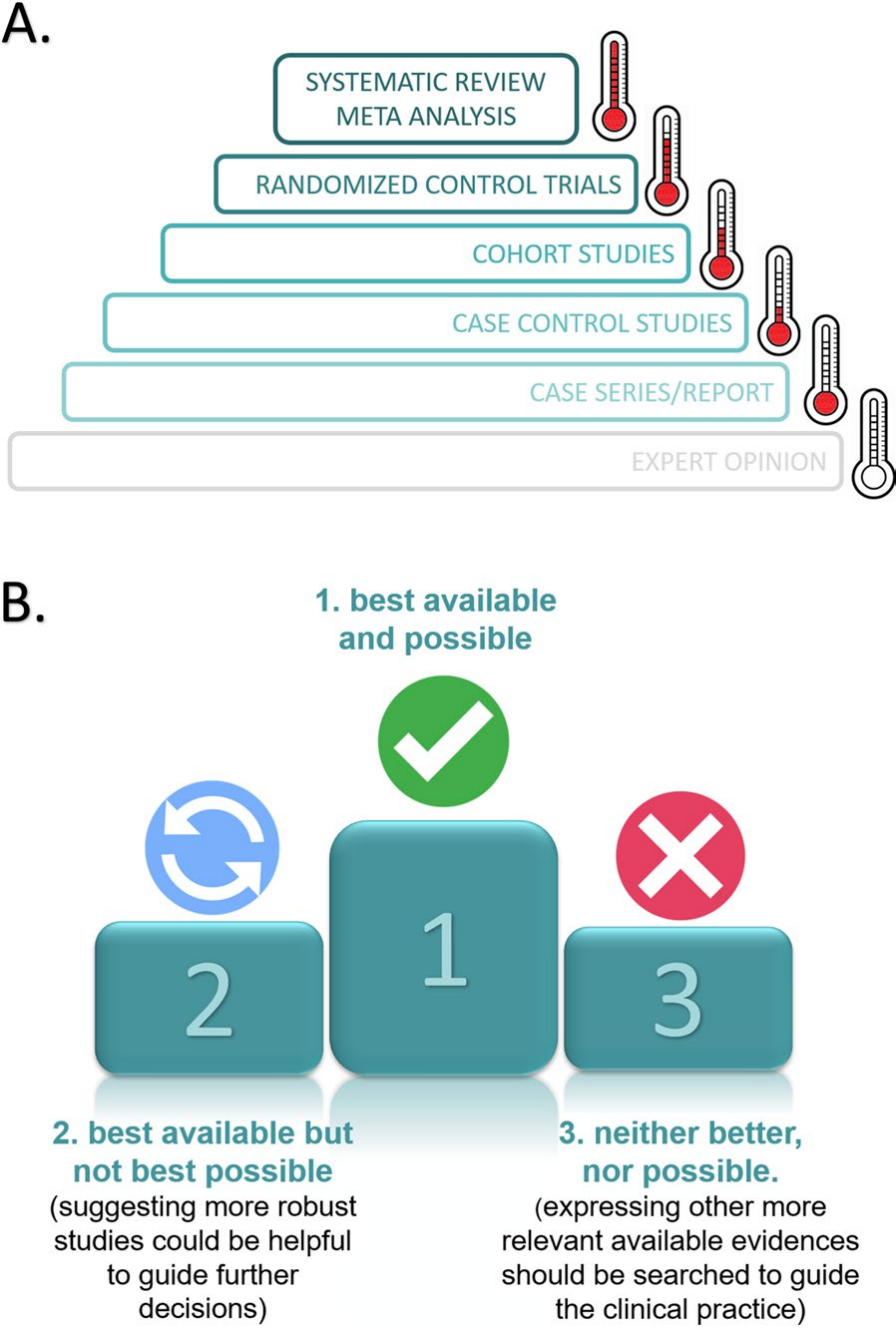
Symbols used to help in the digestion of evidence to clinicians. **A** Thermometer symbol illustrates the strength of study design; **B** representation of available evidence regarding other possible designs (weighing what is possible to do or design and what is currently available). For a more detailed explanation, see Additional file 2

#### Time frame

We consider the first topics available on the page (a pilot cycle) for the present assessments (Additional file 1). This cycle began in March 2015, and posts were, on average, biweekly published. The page and posts were initially promoted by the EviDent team and members of the proponent department, and then, the spread was organic, and no paid tools were used to promote them.

#### Metrics for analysis

Usability metrics (how the page works to users) were collected from Facebook and analyzed to evaluate the initiative's impact 1 year later than its initial exposure to the users. As the pilot cycle was initiated in March 2015, data gathered by Facebook was then collected in March 2016.

As posts on social media could effectively be seen or just exposed to the user, we used different metrics provided by Facebook to assess these different behaviours. The estimated reach means the number of users potentially exposed to the post [18]. Reactions and clicks informed the users' involvement with the page and were used to proxy users' actual access to the posts. As different posts were delivered on different occasions according to the page schedule, the actual exposure time for each post was registered and considered.

Based on these primary metrics, we estimated the user's involvement with the posted content (percentage obtained when dividing the involvement registered during the page use by those users that the page potentially could reach). Finally, we also estimated the percentage of interaction with posted content (clicks in figure and links) by dividing the number of clicks into links or figures by the number of approvals per post. Each one of these metrics will be detailed in Additional file 4.

#### Analysis

Univariate bootstrapped quantile regression analyses (×10,000 and 50% quantile estimation) tested the relationship between the reach and different users' involvement with this content. The order among posts was also tested as an independent variable. The significance level was set as 5%.

### Results

The Facebook page followers reached 1347 up to April 6, 2016, mainly composed of women (88%). Different nationalities were reached, comprising a majority of Brazilian users. The posts exposure time was approximately 11 months (335 days). The estimated average reach was 4784 users per publication (Table 1). The hottest topic received more than 700 reactions and almost 40 accesses on the provided links. The posts order did not impact on user's reach (*P* = 0.99) or involvement (*P* = 0.90). The same trend was observed for the exposure time (P = 0.98 and 0.88, respectively).

**Table 1:** Facebook page usage metrics in chronological order of the posts

Post^a^	ET^b^	Reach^c^	Approval (Like)	Comment	Share	Tr^d^	Fig^e^	Links^f^	Extra^g^	Tc^h^	I^i^	It^j^	ALIt^k^	AFIt^l^
S	385	3042	203	5 (0*)	105	313	121	5	30	156	469	20%	2.45%	59.7%
D	375	3800	186	12 (0*)	35	233	162	17	184	363	596	20%	9.1%	87.1%
E	365	1901	101	8 (1*)	24	133	58	23	50	131	264	18%	22.8%	57.4%
H	333	4080	239	28 (3*)	67	334	108	14	245	367	701	20%	5.7%	45.2%
P1	325	2973	155	6 (2*)	46	207	38	2	116	156	363	14%	1.3%	24.5%
EB	290	2025	87	10 (1*)	26	123	59	12	66	137	260	16%	13.9%	67.8%
P2	277	15,664	556	29 (0*)	129	714	302	38	1356	1696	2410	18%	6.8%	54.3%
Mean (SD)	335.7 (41.8)	4783.5 (4505.2)	218.1 (146.8)	14.0 (9.4)	61.7 (38.0)	293.9 (187.0)	121.1 (83.9)	15.9 (11.1)	292.4 (430.8)	429.4 (526.0)	723.3 (713.0)	18%	7.3%	55.5%

^a^Post: Dental eruption symptoms (S); Interproximal radiographs (D); Resin infiltration (E); Malocclusion (H); Brushing (P1); Sealants (P2); Evidence-based (EB)

^b^Exposure Time (ET)—measured in days since the post was become available

^c^Reach: Number of users who were exposed to the post (in their home feed, for example). It does not mean that they read the content

^d^Total Reactions (Tr): composed of approval, comments, and shares. Reactions that had been deleted were not considered

^e^Figures (Fig): Clicks on figures

^f^Link clicks: Clicks on the links available in the publication. According to Fig. 1, in our work, we usually provide two links: full article and explanation for clinical practice

^g^Extra clicks: other possible clicks different from links and figures, e.g. on the other hyperlinks in the posts, hide, see more, etc.

^h^Total clicks (Tc): the sum of all clicks on the post, be it content, photo, or links available

^i^Involvement (I) = Reactions + clicks

^j^Interaction (It) = Involvement divided by reach

^k^Actual Link Interaction (ALIt): Clicks on link divided by approvals

^l^Actual Figure Interaction (AFIt): Clicks on figure divided by approvals

^*^Administrator’s comments—total: 7

Users’ involvement with page content (Coef = 0.16; 95% CI 0.08–0.23, *P* = 0.003) and clicks on the post (*P* = 0.048; Coef = 0.13; 95% CI 0.001–0.27) were associated with the reach magnitude. The user's interaction/post corresponded, on average, to 18%. Besides, very few clicks to the original article or explanations about the study's relevance were observed. These specific links available in the posts were accessed, on average, 16 times per post.

The reactions to the posts reached approximately 300 marks/publications. On average, 6% of reached users approved (clicked on the "like" button) or shared the post, and the estimated reach of the page was not associated with their users' reaction (*P* = 0.25; Coef = 0.04; 95%; CI − 0.03 to 0.12). Even considering the users that effectively reacted to the posts (likes), only 7%, on average, interacted with the links available in the post to go more in-depth to the topic. Conversely, the actual interaction with figures related to posts reached approximately 60% per post.

### Discussion

One year-long gathered metric indicates that using a Facebook page may have been an interesting way of disseminating digested scientific content rapidly and globally [16, 19]. Similar initiatives also recognize social media as an effective approach for sharing health evidence (increase in followers and/or traffic) among a geographically diverse audience [16, 19–21]. The number of followers of our Facebook reached more than 1300 followers in 1 year of follow-up, reinforcing their interest in receiving the content. Even being an estimated metric, the potential reach observed corroborates the potential of digested scientific evidence as offered to be significantly spread among social media users. However, the users' involvement was lower than expected.

The users' reactions were not associated with the reach metric. Being exposed to more people does not seem to make more people automatically interact with the post content. Indeed, the impact of sharing scientific evidence using social media may not be related to the social media reach [22]. The reactions to the post might be more related to users' preferences and not necessarily to its acknowledged utility [19, 23]. Randomized controlled studies also do not show page access differences between articles promoted through social media, independently of the frequency of dissemination promotion [24, 25].

A low interaction with the posted contents was observed. The interaction with the content posted on our initiative occurred mainly through the approval and endorsement of the page to other potential users. Despite the variations between similar initiatives, different studies [16, 19–21] observed a great reach of the post/tweet and a low interaction with the content disseminated. It is still unclear how social media posts should be structured to optimize their uptake among the target audience [20]. Using other content formats than text (video, infographics, figures) could increase the user interaction with the post [5, 20, 21, 26]. Indeed, 60% of our users interact with figures.

One central differential of our initiative is encouraging clinicians to become protagonists in their evidence-based decisions, which require reading in detail and critically appraisal of the original evidence. The passivity may be seen as a user's proof of reliance on the available content or the clinician's restriction of their learning and decision-making to information digested by others. This last aspect may be an outstanding deal, especially considering the importance of professionals' autonomy and awareness in judging evidence [17]. Despite the timeframe and occasion of data collection, we believe these findings reflect the social media users' interaction profile, and it may be interpreted as an atemporal finding. Policies stimulating such professional behaviour can be created and implemented during professional formation and practice, endorsed by universities, class entities, employers, and different government spheres.

**Fig. 2:**
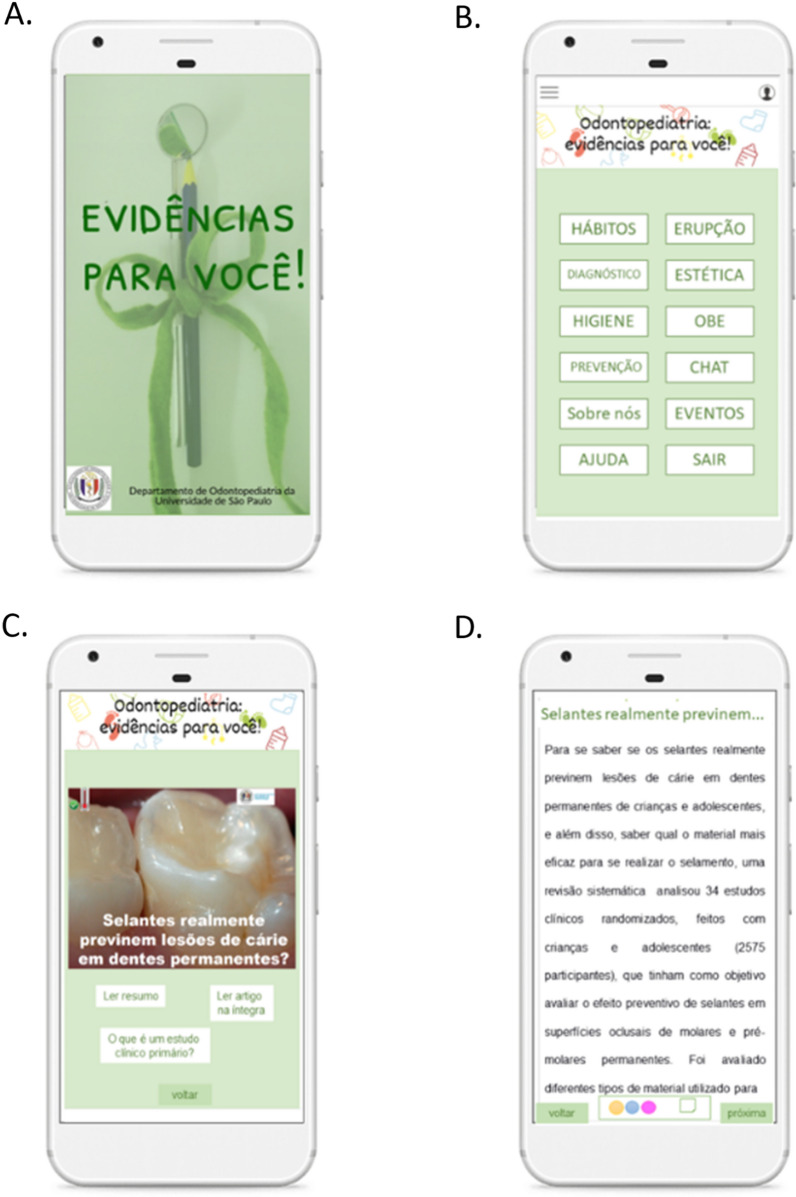
Prototype mApp design development. **A** Splash screen (initial screen of the mApp); **B** main topic menu screen; **C** topic post screen; **D** brief digested content screen. VERSION 0.0 (prototype). The app has been developed in partnership with the Institute of Mathematics and Statistics at the XP Laboratory Course

Based on the barriers above, we have also explored a more proximal alternative to minimize gaps observed when implementing the studied strategy. We have been working on a more interactive and dynamic platform with more engaging content to overcome users' passivity observed. Mobile applications (mApp) are feasible, with high acceptability for health professionals' routines [18, 19] and may permit exploration of different content presentations. The EviDent mApp has been developed (Fig. 2) [27] and will soon be compared to this first initiative. Furthermore, presenting the digested evidence through infographics makes the content user-friendlier than just textual content [21, 26].

In conclusion, although social media has effectively disseminated scientific content, our experience revealed the user's passivity in interacting with the content. This finding seems to be a social media users' pattern, which we expect to overcome by developing a more interactive and attractive interface.

### Limitations

The initiative was designed by pediatric dentists who planned to disseminate the best available evidence in Pediatric Dentistry. Due to this published content, we believe our target audience is dentists and dental professionals. Nevertheless, since our data was collected from organic engagement from an open Facebook page, we cannot assure that. Therefore, we avoid addressing our conclusions focused specifically on dentists.

On the other hand, we do not believe we have reached all Brazilian pediatric dentists. Even if all users are pediatric dentists, our reach would still be lower than those found in Brazil (3540 pediatric—source: Brazilian Federal Dental Council). Therefore, we reinforce we could not generalize our findings to all pediatric dentists, and this is a preliminary appraisal of how the proposed initiative may work on social media.

We opted to use Facebook metrics. Then, some limitations were pointed out to metrics interpretations. Although reactions and clicks proxied the users' involvement with the page, several users could have effectively seen the post but not interacted with it. We adopted a more conservative approach considering the users' involvement as those actions that the page provider could measure during the page use.

As approval is a single interaction metric (signal the user, at least, saw the post), we used it as a reference to create the "actual interactions" metrics. However, this metric may be interpreted cautiously since some users may have explored page content and did not necessarily approve it. It is also essential to address that the actual interaction with figures may be overestimated since users could access the page by clicking on the figure, regardless of the approval.

Social media strategies' effectiveness has been assessed by the number of clicks/impressions on posts, frequency of viewed posts, volume of comments, and replies [28], validating the choice of the used metrics, despite their possible limitations discussed above. These metrics seem more "natural" since users did not acknowledge they were being assessed, minimizing possible "forced" behaviours. Therefore, we may trace initial impressions about the use patterns related to the initiative and permit further initiative development.

## Supplementary Information

**Additional file 1. **EviDent Facebook Page—Topic selection process and those topics selected for the pilot cycle.

**Additional file 2. **EviDent Facebook post structure.

**Additional file 3. **EviDent symbols to the "digestion" of scientific contents on its Facebook page.

**Additional file 4. **Detailed metrics used for assessing Facebook page usability and users' interaction with the contents.

## Data Availability

The datasets regarding Facebook metrics used and/or analyzed during the current study are included in this published article and found in Table 1. The datasets regarding users' profiles (age, sex, nationality, language) are available from the corresponding author on reasonable request.

## Abbreviation

mApp: Mobile applications
