# Supplementary material for: Users' passivity in accessing digested scientific evidence through social media: cross-sectional insights
Source: BMC Res Notes. 2022 Jun 23;15:218. doi: 10.1186/s13104-022-06089-x (PMC9229917; doi:10.1186/s13104-022-06089-x)
Supplement: Supplementary file 1 — Additional file 1. EviDent Facebook Page—Topic selection process and those topics selected for the pilot cycle. [file 13104_2022_6089_MOESM1_ESM.docx]

**Additional File 1****. EviDent Facebook Page – Topic selection process and those topics selected for the pilot cycle.**

***Process:*** *A brainstorming session was conducted, and then topics were defined.* The staff performed a non-systematic literature search to identify each topic's currently best available evidence. The lead researchers were responsible for double-checking the piece of evidence to be scientifically "digested".

**Pilot cycle**

| Topic | Title | Study object | Type of Study |
| --- | --- | --- | --- |
| Eruption | Are there signs or symptoms of dental eruption? (S) | Factors associated with a condition | Longitudinal  (cohort study) |
| Diagnosis | Interproximal radiographs for the detection of carious lesions in primary teeth: what is its real use? (D) | Diagnostic accuracy | Cross-sectional study of diagnostic accuracy |
| Esthetics | Resin infiltration: A new option in the aesthetic approach to initial caries lesions? (E) | Intervention efficacy | Randomized controlled trial for intervention |
| Habits | Is there a relationship between bottle use and malocclusion in primary dentition? (H) | Exposure factors associated with a condition | Systematic review of observational studies |
| Prevention | Is the extra-long-bristled toothbrush a viable alternative to plaque removal when brushing occlusal surfaces of erupting permanent molars? (P1)  Do Sealants Really Prevent Caries Injuries to Permanent Teeth? (P2) | Intervention efficacy and costs  Intervention efficacy and costs | Randomized controlled trial for intervention and economic evaluation  Systematic review of randomized controlled trials |
| Evidence-based Practice | Evidence-based practice? what is it? (EB) | Opinion – editorial | Expert’s opinion |
