# Supplementary material for: Users' passivity in accessing digested scientific evidence through social media: cross-sectional insights
Source: BMC Res Notes. 2022 Jun 23;15:218. doi: 10.1186/s13104-022-06089-x (PMC9229917; doi:10.1186/s13104-022-06089-x)
Supplement: Supplementary file 3 — Additional file 3. EviDent symbols to the "digestion" of scientific contents on its Facebook page. [file 13104_2022_6089_MOESM3_ESM.docx]

**Additional File 3. EviDent symbols to the "digestion" of scientific contents on its Facebook page**

Symbols were used on each post to help clinicians understand digested evidence's relevance. The first symbol was a thermometer, a manner we chose to express the strength of the evidence (Figure 1). We limited the judgement to the study design chosen to answer a clinical question to simplify this task. This symbol is linked to strengths and weaknesses from different types of studies, similar to the idea from the traditional evidence pyramids for classifying evidence related to interventions (1). Analogously, the higher the temperature is shown in the thermometer, the greater the study design relevance to answering that research question and guiding clinical practice.

The traditional pyramid of evidence has been extensively used in evidence-based practice. Nevertheless, it is often indigestible to clinicians, who usually fail to identify methodological aspects in the paper, as the study design.

As the maximum level of evidence traditionally presented in the hierarchy pyramids is mainly focused on interventional studies, clinicians also get confused about which type of study would be more trustful when other issues are explored. Besides, some limitations in the study design may also impact their certainty to guide clinical practice (2). That is why another symbol was created to classify the evidence weighing what is possible to do or design, and what is currently available. A scale was created to differentiate all possible situations (Figure 1B). 1. best available and possible evidence, optimal situation (checkmark symbol), 2. best available but not best possible evidence, suggesting more robust studies could be helpful to guide decisions further (loading symbol); or 3. neither better evidence available nor possible evidence, expressing other more relevant available evidence should be searched and chosen to guide the clinical practice ("x" mark symbol).

Figure 1 – Symbols used to illustrate the evidence relevance presented in the post. A- Thermometer symbol illustrating the strength of study design; B- Representation of
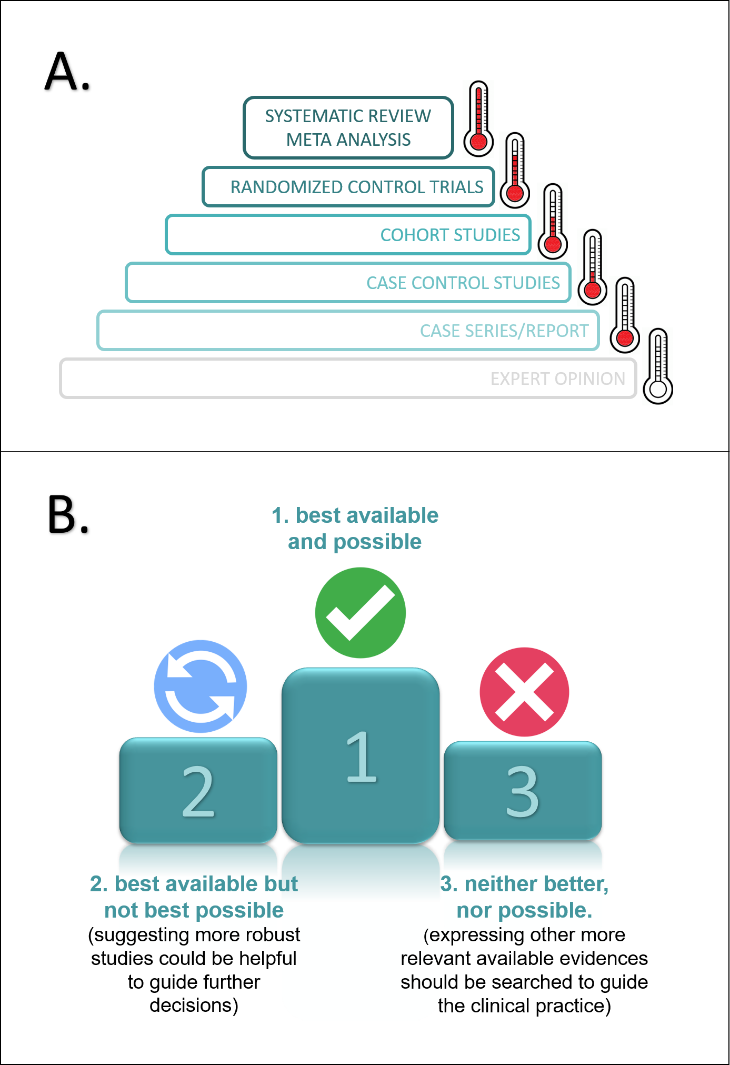
available evidence regarding other possible designs (weighing what is possible to do or design and what is currently available)

References

1. Stegenga J. Down with the Hierarchies. Topoi. 2014;33(2):313-22.

2. Atkins D, Best D, Briss PA, Eccles M, Falck-Ytter Y, Flottorp S, et al. Grading quality of evidence and strength of recommendations. BMJ (Clinical research ed). 2004;328(7454):1490-.
