# Supplementary material for: Users' passivity in accessing digested scientific evidence through social media: cross-sectional insights
Source: BMC Res Notes. 2022 Jun 23;15:218. doi: 10.1186/s13104-022-06089-x (PMC9229917; doi:10.1186/s13104-022-06089-x)
Supplement: Supplementary file 4 — Additional file 4. Detailed metrics used for assessing Facebook page usability and users' interaction with the contents. [file 13104_2022_6089_MOESM4_ESM.docx]

**Additional File** **4.** **Detailed metrics used for assessing Facebook page usability and users' interaction with the contents**

Metrics

*Facebook Metrics*

- Potential Page Exposure to users

**Reach:** The reach represents the number of users exposed to the post, which means the users who may have viewed the post at least once. This metric is estimated by Facebook using a representative sample of users. This figure is estimated considering the user could view the post, but not necessarily they accessed the full post or all page (Facebook). Therefore, it is independent of the user's intentional action.

- Users' interaction

**Reactions:** it is a metric including user's approval, comments, and shares, representing the actual count of the user's activity related to any specific post. The approval represents the number of likes received in that post. In other words, how many users thought that post was good. For this reaction, only one interaction per user was possible. Besides, shares quantify how many times users have shared that post, and comments represent the number of comments received in that post. These two last types of reactions permit more than one interaction per user and also include administrator's interventions (e.g. answer to users' comments or queries; shares with close pairs). All these metrics are dynamic (they can be changed or removed/deleted deliberately by users (even for administrators, for the comments). The actual figures at the moment of data collection were considered.

**Clicks:**  it is a metric related to the user's interaction on hyperelements (links, figures, etc.) included in the post. The actual number of clicks was registered since it is impossible to undo a click. Thus, we can analyze all clicks on the post (and on the page in which it is inserted). We can also assess the clicks on links and figure separately. For our page, the available links could guide the user to the full article (after reading the brief digested content) or to the explanation about the type of study. Using this metric, we could estimate the users' interest in going deeper into the content/topic. We had only one figure per post and then clicked to expand the post revealing the full post (brief digested content and links). In addition to these clicks, the metric "other clicks" also includes when the users click on any other media, like the "see more" to expand the post, or "enter full screen", or some tag in the figure, etc.

*Secondary Research Metrics*

Considering the metrics above provided by Facebook, we calculated the user's involvement and estimated the interaction with the post, as described below.

**Involvement:** The user's involvement was calculated as the sum of reactions and clicks (reactions + clicks). It considers the two metrics dependent on the user's action (involvement) with the post.

**Interaction percentage:** We calculated the percentage of user's interactions per post by dividing the involvement by the reach (number of users possibly exposed to the post). Then, we could assess users that actively interacted with the post among those who could have seen it in their feeds.

**Actual Link and Figure Interaction percentage:** For estimating this metric, we consider the number of approvals as the number of users that effectively access the post (not being only exposed to them). This metric was chosen because it is the one that provides an actual single count per user, permitting a closer estimation of interaction than when the user's reach was considered. Then, we calculated the actual interactions based on this parameter.

The actual link interaction was calculated by dividing the number of clicks on links by the number of approvals per post. In contrast, the actual figure interaction was given the ratio between the number of clicks on figures and the number of approvals per post.
